# Adherence to Highly Active Antiretroviral Therapy Among Children in Ethiopia: A Systematic Review and Meta-analysis

**DOI:** 10.1007/s10461-018-2152-z

**Published:** 2018-05-14

**Authors:** Aklilu Endalamaw, Nega Tezera, Setegn Eshetie, Sintayehu Ambachew, Tesfa Dejenie Habtewold

**Affiliations:** 1 Department of Pediatrics and Child Health Nursing, School of Nursing, College of Medicine and Health Sciences, University of Gondar, P.O. BOX: 196, Gondar, Ethiopia; 2 Department of Medical Microbiology, School of Biomedical and Laboratory Sciences, College of Medicine and Health Sciences, University of Gondar, Gondar, Ethiopia; 3 Department of Clinical Chemistry, School of Biomedical and Laboratory Sciences, College of Medicine and Health Sciences, University of Gondar, Gondar, Ethiopia; 4 University Center for Psychiatry, Rob Giel Research Centre, University of Groningen, University Medical Center Groningen, Groningen, The Netherlands; 5 Department of Epidemiology, University of Groningen, University Medical Center Groningen, Groningen, The Netherlands

**Keywords:** Adherence, Antiretroviral therapy, Children, Highly active, HIV, Medication, Meta-analysis, Ethiopia, Adherencia, Terapia antirretroviral altamente activa, Niños, VIH, Medicamentos, Meta-análisis, Etiopía

## Abstract

Adherence to highly active antiretroviral therapy (HAART) is the mainstay of the strategy in reducing morbidity and mortality of HIV-infected children. Different primary studies were conducted in Ethiopia. Thus, we aimed to conduct a meta-analysis of the national prevalence of optimal adherence to HAART in children. In addition, associated factors of HAART adherence were reviewed. A weighted inverse variance random-effects model was applied. The 88.7 and 93.7% of children were adhering to HAART at 07 and 03 days prior to an interview respectively. The subgroup analysis showed that HAART adherence was 93.4% in Amhara, 90.1% in Addis Ababa and 87.3% in Tigray at 07 days prior to an interview. Our study suggests that, within short window reported time, adherence to HAART in Ethiopian children may be in a good progress. Emphasis on specific adherence interventions need further based on individual predictors to improve overall HAART adherence of children.

**Electronic supplementary material:**

The online version of this article (10.1007/s10461-018-2152-z) contains supplementary material, which is available to authorized users.

## Resumen

La adherencia a la terapia antirretroviral altamente activa (HAART) es el pilar de la estrategia de reducción de la morbilidad y la mortalidad de los niños infectados con el VIH. Diferentes estudios primarios fueron realizados en Etiopía. Por lo tanto, decidimos realizar un meta-análisis de la prevalencia nacional de excelente adherencia al TARGA en los niños. Además, los factores asociados de cumplimiento del TARGA fueron revisados. Inverso de la varianza ponderada de un modelo de efectos aleatorios se aplicó. El 88,7% y el 93,7% de los niños estaban adheridos al TARGA en 07 y 03 días antes de una entrevista, respectivamente. El análisis de subgrupos demostró que el cumplimiento del TARGA fue de 93,4% en Amhara, 90,1% en Addis Abeba y 87,3% en Tigray en 07 días previos a la entrevista. Nuestro estudio sugiere que, dentro de breve tiempo informado de la ventana, la adherencia al TARGA en niños etíopes pueden tener un buen progreso. Énfasis en la adherencia al tratamiento, las intervenciones específicas deben seguir basándose en predictores individuales para mejorar el nivel general de cumplimiento del TARGA de niños.

## Background

According to 2017 World Health Organization’s (WHO) report, 36.7 million people were living with HIV/AIDS (Human Immunodeficiency Virus/Acquired Immunodeficiency Syndrome) around the globe, of which 2.1 million were children less than 15 years of age [1]. It is estimated 1.1% of the Ethiopian population living with HIV, of which 10% were children [2]. Access to highly active antiretroviral therapy (HAART) in low- and middle-income countries has expanded dramatically [3]. Subsequently, HAART coverage is increasing; and nearly 20.9 million people were taking HAART in June 2017. Among children living with HIV, 43% accessed HAART [1]. In Ethiopia, 61% of adults aged 15 years and older living with HIV had access to HAART, but just 33% of children aged below 15 years had access [2].

HIV-related morbidity and mortality occurred significantly even in the presence of HAART [4–7] although HAART plays a significant role in improving the life of HIV-positive patients [8]. Adherence to drugs is critical in determining the efficacy and durability of HAART regimens [9, 10]. For optimal therapeutic effect, at least ≥ 95% HAART adherence has been suggested [11, 12], though the minimum value for optimal adherence is not well defined [13, 14]. Missing a prescribed regimen has a remarkable contribution to the emerging of treatment failure [15–17] and resistance to HIV/AIDS drugs [18, 19].

Adherence promoting-interventions are implementing in different parts of the world including Ethiopia. Notably, implementing interventions evaluation, home-based HAART [20], social support [21, 22], educational [23], daily observable treatment [24], and community-based HAART program [25] are considering the main strategies to increase optimal HAART adherence of HIV-infected children.

Although measuring of optimal adherence remains a challenge since there is no single method that is reliable [26, 27], the most frequently used measure of adherence in children is caregivers’ report. Accordingly, the prevalence of optimal adherence of children to HAART is 90.9% in South India [28], 77% in India [29], 77% in China [30], 42% in West Africa [31] and 76.1% in Nigeria [32].

Common barriers to HAART optimal adherence are categorized as socio-demographic, behavioral, clinical and health system related factors [28, 33, 34]. Among these factors, some of them are unfavorable school environment, pills burden of the HIV drug, treatment longevity, being unaware of HIV status, non-parental care, preference for traditional medicine and forgetfulness [35–38].

National [39, 40] and international [41–44] systematic review and meta-analysis have been conducted among the adult population. In Ethiopia, many studies [45–55] have been conducted on the prevalence of optimal adherence to HAART and its associated factors among HIV-infected children. Discrepancies among studies in the same geographical area, across regions, at a similar and different time period, were reported. While the study by Dachew et al. [47.] estimated a high rate of 96.8% HAART adherence, the study by Feyissa [54] showed a prevalence of 61.5%. Therefore, the national prevalence of optimal adherence to HAART and its contributing factors among HIV-infected children are poorly understood. Given the above gaps, the aim of the present study was to (i) estimate the national pooled prevalence of optimal adherence to HAART from the available literature of HIV-infected children in Ethiopia that maintain an intake of ≥ 95% of prescribed HAART and [4] systematically review the associated factors of HAART adherence in HIV-infected children. The finding of this study will provide information for clinicians, policy and decision makers.

## Methods

### Reporting

The Preferred Reporting Items for Systematic Reviews and Meta-analysis (PRISMA) guideline was used to report the finding of this review [56] (Additional file research checklist). The protocol of this systematic review and meta-analysis was registered in the Prospero database: (PROSPERO 2017: CRD42018081755).

### Databases and Search Strategy

A comprehensive search was carried out in PubMed, Google Scholar, Web of Science, Wiley Online Library and EMBASE electronic database up to 03 November 2017. The search focused on the studies with reported prevalence of optimal HAART adherence and/or at least one associated factor among Ethiopian children. The search further limited to articles published in English. The following terms and/or phrases were used: “ART”; “HAART”; “Antiretroviral therapy”; “highly active antiretroviral therapy”; “adherence”, “poor adherence”; “good adherence”; “non-adherence”; “patient compliant”; “children”; “child”; “Pediatrics”; “infant”; “infants”; and “Ethiopia”. Search strings were implemented using “AND” and “OR” Boolean operators.

### Inclusion and Exclusion Criteria

The studies were included if they met the following inclusion criteria: [1] studies conducted on children < 15 years of age; [2] observational studies, including cross-sectional, cohort and case–control studies; [3] studies that reported prevalence of optimal adherence and/or at least one predictor, which was adjusted to other factors; [4] the outcome was optimal adherence to HAART; [5] studies conducted in Ethiopia; [6] studies published in English language. Optimal adherence to HAART among included studies in this meta-analysis was defined as according to caregiver report if children took ≥ 95% of the prescribed doses for 07 days [45–54] and 03 days [46, 47, 49, 50, 53, 55] prior to an interview. Qualitative studies and citations without full-text were excluded. Studies conducting on adherence to HAART prophylaxis like mother to child transmission prophylaxis were also excluded.

### Study Selection and Quality Assessment

We used Endnote version 7 (Thomson Reuters, London) reference manager to remove duplicated studies. Two reviewers (AE and TD) independently screened the titles and abstracts to consider the articles in the full-text review. Two investigators (AE and NT) assessed the quality of the studies using Joanna Brigg’s Institute quality appraisal criteria (JBI) [57]. The following items were used to appraise the selected studies: [1] inclusion criteria; [2] description of study subject and setting; [3] valid and reliable measurement of exposure; [4] objective and standard criteria used; [5] identification of confounder; [6] strategies to handle confounder; [7] outcome measurement; and [8] appropriate statistical analysis. The disagreement was solved by consensus. Studies got 50% and above of the quality scale were considered low risk.

### Data Extraction

Two independent reviewers (AE and SE) extracted the data. The procedure was repeated whenever inconsistency occurred. Information about the first author and year of publication, study setting, study design, sample size, and prevalence and associated factors of optimal adherence to HAART were extracted. Assessment method for optimal adherence in all included studies was through caregiver report. Studies preferred times to report the prevalence of optimal adherence were 07 and/or 03 days prior to an interview. All included studies didn’t consistently use both 03 and 07 days prior to an interview. To answer the questions regarding [1] the prevalence of optimal adherence at 07 days prior to an interview and [2] the prevalence of optimal adherence at 03 days prior to an interview, we collected the data in the theme, in which studies reported the prevalence of optimal adherence at 07 days as one theme and studies reported the prevalence of optimal adherence at 03 days in the other theme.

### Data Analysis

A weighted inverse variance random-effects model [58] was used to estimate the prevalence of optimal HAART adherence at 07 and 03 days prior to an interview. The variation in the pooled estimates of the prevalence was adjusted through subgroup analysis according to the region, where the study conducted. Heterogeneity across the studies was assessed using I^2^ statistic where 25, 50 and 75% representing low, moderate and high heterogeneity respectively [59]. A Funnel plot and Egger’s regression test were used to check publication bias [60]. A sensitivity analysis was conducted to check the stability of summary estimate. We used STATA version 14 (Stata Corp, 4905 Lake way Drive, College Station, Texas 77845 USA) statistical software to conduct this meta-analysis.

## Result

### Characteristics of Included Studies

**Fig. 1:**
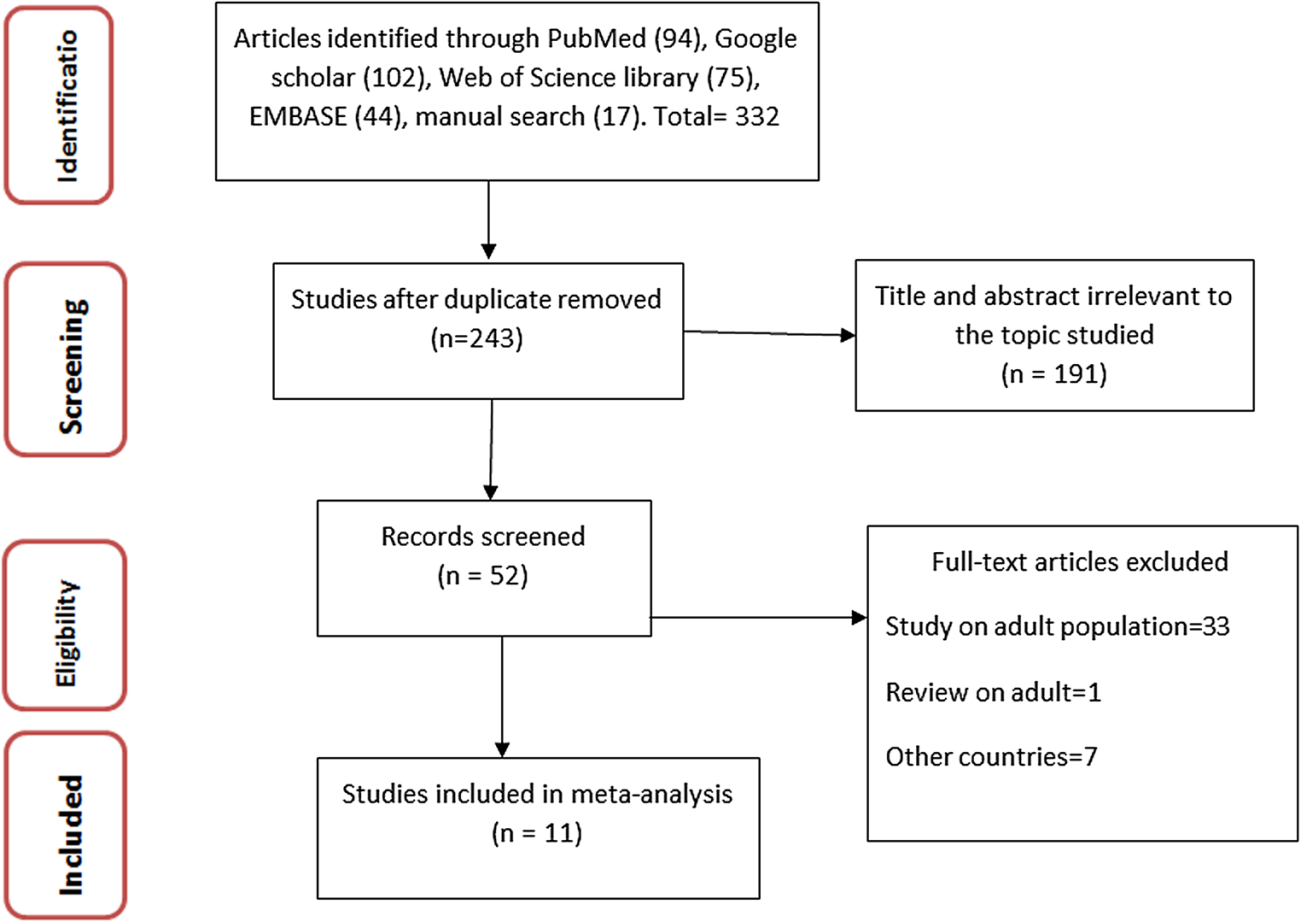
PRISMA flow diagram showed the results of the search and reasons for exclusion

In total, 332 potential studies were identified; 94 articles from PubMed, 102 articles from Google Scholar, 44 from EMBASE, 75 articles from Web of Science, and 17 articles through manual search. Figure 1 showed the results of the search and reasons of exclusion during the study selection process. A total of 11 studies were included to assess the prevalence of optimal adherence to ART at 07 and 03 days prior to an interview.

Included studies were published between the years 2008 and 2017. A cross-sectional study design was employed for all included studies. Three studies conducted in Amhara region [46, 47, 55], two in Oromia [52, 54], two in Tigray [50, 53], two in Addis Ababa [45, 48], one in Oromia and Addis Ababa [51] and one in Harare and Dire Dawa city [49]. Five studies reported the prevalence of optimal adherence both at 07 days and 03 days prior to an interview [46, 47, 49, 50, 53] whereas five studies reported only prevalence at 07 days prior to an interview [45, 48, 51, 52, 54]. One study reported the prevalence of optimal adherence at 03 days prior to an interview [55]. Therefore, ten studies [45–54] reported the prevalence of optimal adherence at 07 days and six studies [46, 47, 49, 50, 53, 55] at 03 days prior to an interview. A total of 2,864 HIV-infected children were participated in the included studies. The minimum sample size was 120 observed in Oromia [52, 54] and the maximum 440 in Amhara region [46]. Table 1 presents the characteristics and outcomes of reviewed studies.

**Table 1:** General characteristics and outcomes of the included studies (n = 11)

Author/year	Study area	Study design	Sample size	Prevalence at 07 days prior to interview	Prevalence at 03 days prior to interview	Quality
Biresaw S et al./2013	Addis Ababa	Cross-sectional	210	93.3	_	Low risk
Arage G et al./2014	Amhara	Cross-sectional	440	89.8	95.9	Low risk
Dachew BA et al./2014	Amhara	Cross-sectional	342	96.8	98.7	Low risk
Biadgilign S.et al./2008	Addis Ababa	Cross-sectional	390	86.9	_	Low risk
Zegeye S, Sendo EG/2015	Harare and Dire dawa	Cross-sectional	313	97	99	Low risk
Eticha T, Berhan L/2014	Tigray	Cross-sectional	193	83.4	89.1	Low risk
Biru M et al./2017	Oromia and Addis Ababa	Cross-sectional	306	92.8	_	Low risk
Alemu K et al./2014	Oromia	Cross-sectional	120	84.2	_	Low risk
Gultie T et al./2014	Tigray	Cross-sectional	226	90.7	92.9	Low risk
Feyissa A/2016	Oromia	Cross-sectional	120	61.5	_	Low risk
Azmeraw D, Wasie B/2012	Amhara	Cross-sectional	204	_	80.9	Low risk

### Quality of the Included Studies

All studies were assessed using JBI checklist for cross-sectional studies. The assessment with JBI quality appraisal checklists indicated that none of the included studies were of poor in quality and excluded from the meta-analysis (Table 1).

### Meta-analysis

#### Prevalence of Adherence 07 Days Prior to Interview

**Fig. 2:**
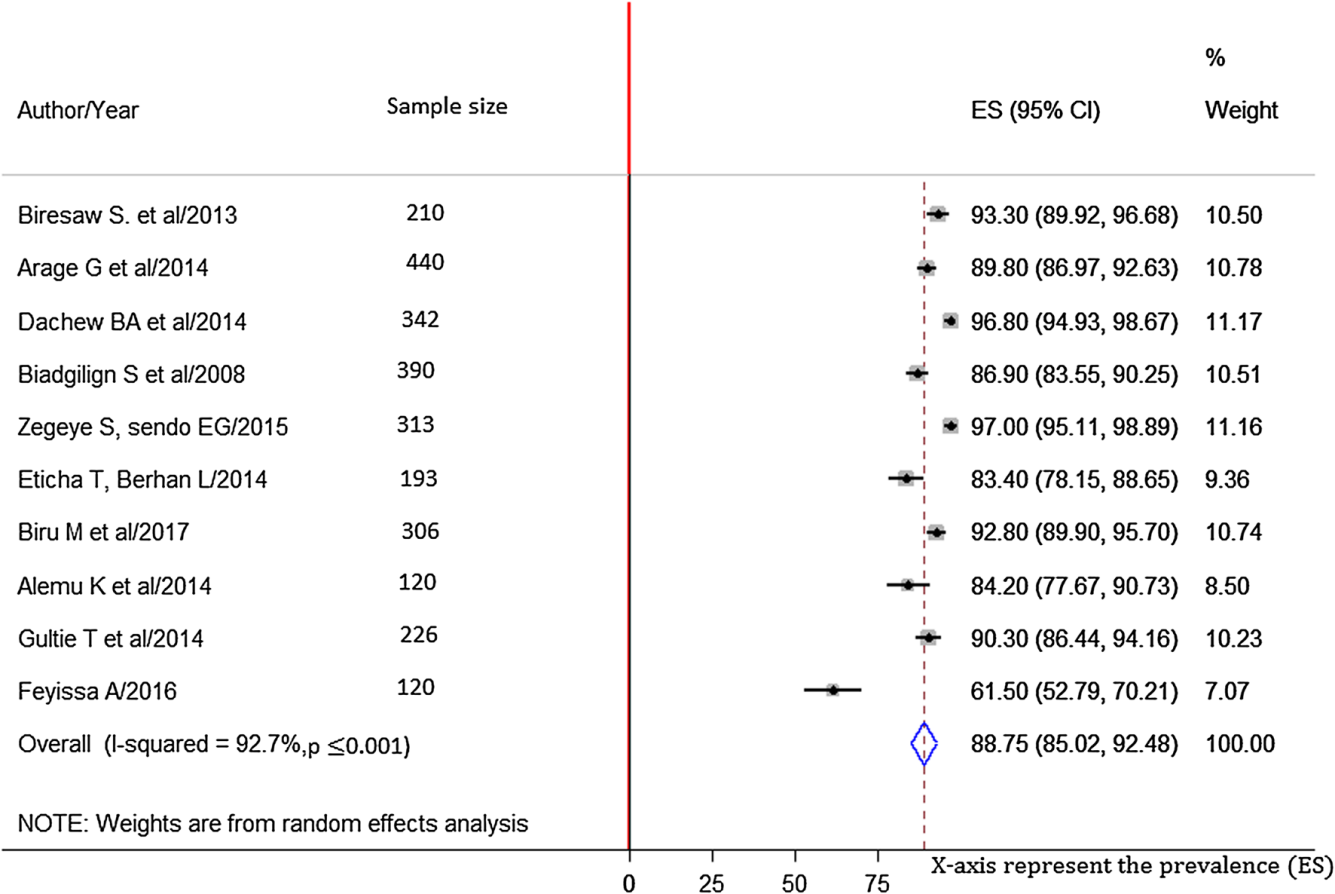
Pooled estimates of the prevalence of optimal adherence to ART among HIV-infected children 07 days prior to the interview. The midpoint and the length of each segment indicated prevalence and a 95% CI whereas the diamond shape showed the combined prevalence of all studies

The prevalence of optimal HAART adherence among HIV-positive children at 07 days prior to interview ranges from 61.5% (95% confidence interval (CI) 52.8–70.2) in Oromia [54] to 96.8% (95%CI 94.9–98.7) [49] in Amhara region. The estimated national pooled prevalence of optimal HAART adherence was 88.8% (95% Confidence Interval (CI) 85.1–92.5, I^2^ = 92.6%; *p* value < 0.001) (Fig. 2).

#### Prevalence of Adherence 03 Days Prior to Interview

**Fig. 3:**
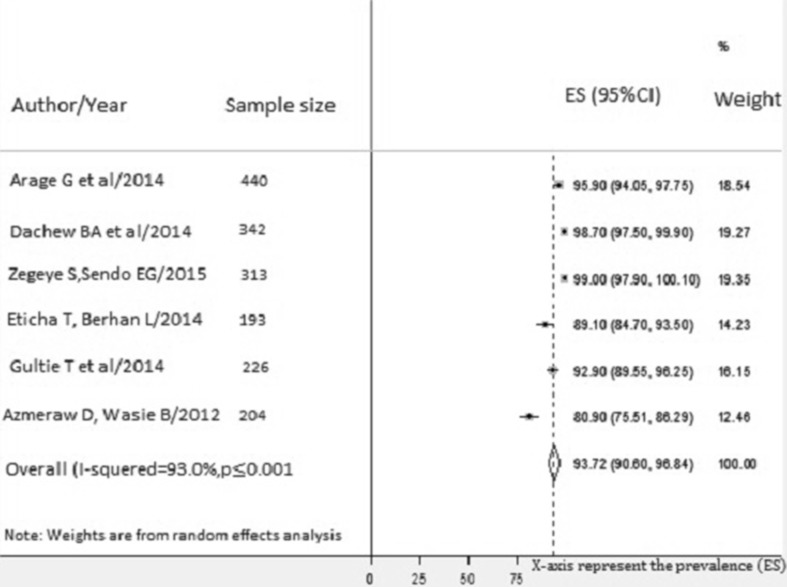
Pooled estimates of optimal adherence of children to ART 03 days prior to an interview. The midpoint and the length of each segment indicated prevalence and a 95% CI whereas the diamond shape showed the combined prevalence of all studies

The prevalence at 03 days prior to an interview ranges from 80.9% (95%CI 75.5–86.3) [55] in Amhara to 99.0% (95%CI 97.9–100.1) Harare and Dire Dawa [49]. The national pooled prevalence of optimal HAART adherence at 03 days prior to an interview was 93.7% (95% CI 90.6–96.8, I^2^ = 93.0%; p value < 0.001) (Fig. 3).

#### Subgroup Analysis

Subgroup analysis based on geographical area (study setting) was estimated. The prevalence at 03 days prior to an interview was 92.7% in Amhara and 93.9% in Harare and Dire Dawa (Additional file Fig. 1). The prevalence at 07 days prior to an interview was 90.1% in Addis Ababa, 93.4% in Amhara, 87.3% in Tigray, 73.0% in Oromia and 95.1% in others region (Additional file Fig. 2).

#### Sensitivity Analysis

We did the sensitivity analysis of adherence to HAART by applying a random effects model (Table 2). Excluded studies with a low number of participants resulted in a slight difference in the prevalence of optimal HAART adherence.

**Table 2:** Sensitivity analysis 07 and 03 days prior to an interview

Study omitted	Prevalence of optimal adherence (95%CI) 07 days prior to an interview	Prevalence of optimal adherence (95%CI) 03 days prior to an interview
Biressaw S. et al./2013	88.2 (84.0, 92.3)	_
Arage G et al./2014	88.6 (84.5, 92.7)	93.0 (89.2, 96.9)
Dachew BA et al./2014	87.7 (83.5, 91.9)	92.2 (87.6, 96.7)
Biadgilign S et al./2008	89.0 (85.1, 92.9)	_
Zegeye S, sendo EG/2015	87.7 (83.5, 91.8)	92.1 (87.7, 96.5)
Eticha T, Berhan L/2014	89.4 (85.6, 93.2)	94.6 (91.5, 97.7)
Biru M et al./2017	88.2 (84.0, 92.4)	_
Alemu K et al./2014	89.2 (85.4, 93.1)	_
Gultie T et al./2014	88.5 (84.4, 92.6)	93.9 (90.5, 97.3)
Feyissa A/2016	91.0 (88.0, 94.0)	_
Azmeraw D, Wasie B/2012	_	95.9 (93.5, 98.3)
Combined	88.8 (85.1, 92.5)	93.7 (90.6, 96.8)

#### Publication Bias

**Fig. 4:**
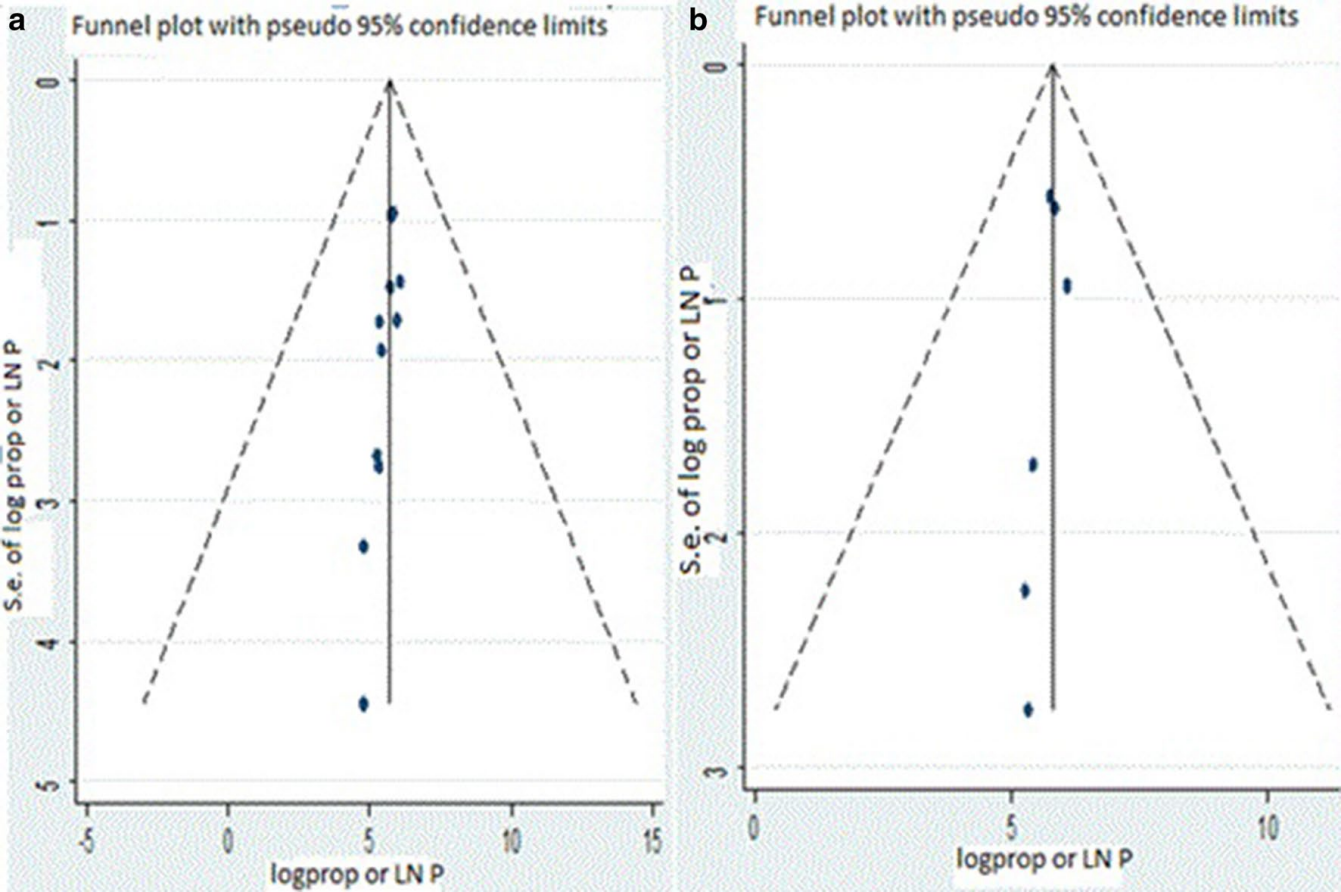
Graph **a** stands 07 days and graph **b** 03 days prior to an interview

We visually examined signs of asymmetry using Funnel plots to assess publication bias (Fig. 4). Moreover, more objectively, Egger’s regression test resulted in a p value = 0.23 and p value = 0.26, 07 and 03 days prior to an interview respectively, which indicates the absence of publication bias in both cases.

#### Factors Associated with Adherence to HAART

This systematic review identified eleven studies that reported factors associated with HAART adherence as categorized into four themes related to (1) children and caregiver socio-demographic, (2) clinical and medication, (3) behavioral, and (4) health care system.

#### Children and Caregiver Demographic-Related Factors

Being in the age group 5–9 years [adjusted odds ratio (AOR) = 0.42 (95% CI 0.36, 0.54)] and 10–15 years [AOR = 0.37 (95%CI 0.31, 0.46)] were less likely to have optimal adherence as compared to 0–5 years old children [47]. In contrary, one study showed that being in the age group below 5 years [AOR = 1.4 (95%CI 1.2, 3.9)] was more likely to adhere as compared to > 10 years age children [53]. Children with male caregiver [AOR = 2.10, 95% CI (1.01, 7.2)] was positively associated with HAART adherence [53]. Other factors associated with suboptimal adherence across studies including being female (AOR = 3.9) [52], children with the age group of 25–34 years (AOR = 22.3 (95%CI 4.3, 114.3) and 35–44 years caregiver [AOR = 7.1 (95%CI 1.6, 30.9)] as compared to those with > 44 years age group [50]. Another study revealed, children with caregivers who had secondary or above educational status [AOR = 0.59 (95%CI 0.21, 0.83)] were less likely adhere with HAART. Children with unmarried [AOR = 15.2 (95%CI 3.4, 68.4)] and married caregiver [AOR = 3.5 (95%CI 1.2, 10.1)] [50] were more likely to have optimal HAART adherence than those with a divorced/separated caregiver. One study also revealed that children with married (AOR = 7.8 (95%CI 2.1, 29.1) and widowed/divorced caregiver (AOR = 7.1 (95%CI 2.0, 25.5) were more likely to have optimal adherence as compared to those with single caregiver [45].

#### Clinical and Medication Related Factors

Children on WHO stage II (AOR = 0.128) and IV (AOR = 0.055) were less likely to optimal HAART adherence as compared to WHO stage I [52]. Other study explained that children on WHO stage III and IV [AOR = 3.2 (95%CI 1.2, 8.4)] were less likely to have optimal HAART adherence as compared to WHO stage I and II [45]. Children who had CD4 count ≥ 500 (AOR = 1.9 (95%CI 1.3, 3.9) were more likely adhere to HAART as compared to < 500 [46]. Regarding types of HAART, children who were on first line HAART drugs [AOR = 2.9 (95%CI 1.5, 3.7)] were more likely adhere to HAART [53]. Children who were on 4b (d4T/3TC/EFV) ART [AOR = 0.1 (95%CI 0.02, 0.53)] were less likely adhere to HAART as compared to those who were on 4a (d4T/3TC/NVP) [45]. Whereas, children received LPV/r or ABC [AOR = 12.3 (95%CI 3.3, 46.7)] were less likely adhere to HAART as compared to those children received 4a/4b or 4c/1c/4d [51]. Children who took Cotrimoxazole besides HAART [AOR = 3.65 (95%CI 1.2, 10.7)] were more likely to have optimal HAART adherence [48]. Children whose caregivers were not undergoing HIV care and treatment themselves were less likely to have optimal HAART adherence (AOR = 0.2, 95% CI 0.04, 0.7) [51].

#### Behavior Related Factors

Among the reviewed studies, three were showed children who were not aware of their HIV sero-status were more likely adhere to HAART as evidenced by [AOR = 3.5 (95%CI 2.1, 6.8)] [46], [AOR = 2.4 (95%CI 1.1, 5.1)] [45], [AOR = 2.5(95%CI 1.2, 5.2)] [48]. Aside from another one study, children aware of their HIV status [AOR = 0.27 (95CI 0.24, 0.32)] were less likely to have optimal HAART adherence [47]. Two studies showed that children with substance user caregivers [AOR = 2.2 (95%CI 1.3, 5.4)] [46], (OR = 0.31, 95%CI 0.10, 0.93) [55] were less likely to HAART adherence. Children whose caregivers did not use a medication reminder (AOR = 5.2, 95% CI 2.2, 12.2) were more likely to HAART non-adherence [51].

Children with caregivers who had good knowledge about HAART (AOR = 4.7 (95%CI 3.7, 5.6) [47], [AOR = 2.7 (95%CI 1.8, 7.1)] [46], [AOR = 7.31(95%CI 1.7, 6.1)] [49] was additional factors found in the review to be promoting factors of optimal HAART adherence.

#### Health Care System Related Factors

Children living in < 10 K.M far distance from the health facility [AOR = 2.3 (95%CI 1.9, 4.6)] were more likely to have optimal HAART adherence as compared to > 10 KM [46]. Children who had ever received any nutritional support from the clinic were 66.3% less likely to adhere with HAART than those who did not get the nutritional support [AOR = 0.34 (95% CI 0.14, 0.79)] [48].

## Discussion

This systematic review and meta-analysis was conducted to estimate the national pooled prevalence of optimal HAART adherence among HIV-infected children in Ethiopia. Besides, significantly associated factors of HAART adherence were systematically reviewed. Accordingly, the national pooled prevalence of optimal HAART adherence at 07 and 03 days prior to an interview was 88.8 and 93.2% respectively.

The result of this meta-analysis was comparable to a study conducted in South India (90.9%) [28]. However, it was higher than a globally meta-analyzed report (62%) [41], China (77.6%) [30], Africa (77%) [61], India (70%) [29], West Africa (42%) [31]. These discrepancies might be due to the difference in socio-demographic characteristics, healthcare systems, the adherence report method and/or date, study population and study design.

The subgroup analysis showed that the adherence of children to HAART in Amhara region (93.4%) was consistent to Addis Ababa (90.1%). In contrast, it was higher than Oromia region (73.04%) and Tigray region (87.3%). This variation might be due to the difference in health care system and clinical setting, attitude and awareness of caregivers about HAART. Additionally, beliefs about the benefit of HAART, religious practices and use of traditional medicine might have an influence on optimal adherence in Oromia region [62]. However, the prevalence of optimal adherence to HAART 03 days prior to an interview was comparable between regions. This might be due to the fact that as the duration of taking drugs increased, the probability of missing drugs would increase due to different reasons.

The children and caregiver, and clinical and medication, behavioral, and health care system related variables were contribute on HAART adherence of children Ethiopia. In agreement with our review, varieties of studies both in developed and developing countries identified such like variables [63–67].

Our systematic review showed that age [47] and sex of children [52], caregivers’ age [50] and marital status [45, 50] were associated factors of HAART adherence. Older children were less likely to have HAART adherence. As children age increased, many responsibilities are given so that children couldn’t successfully handle the treatment regimen. Female children were less likely to adhere to HAART. This might be due to the influence of gender roles, in which they could forget taking of HAART pills per the scheduled. Children whose caregivers were married were more adhere to HAART. Married caregivers might have support from their husband in providing respectful and compassionate care.

Regarding clinical related factors, WHO clinical stage III and IV [45], use of first-line HAART drugs [53], and use of Cotrimoxazole [45] were positively influencing HAART adherence. Those children with advanced opportunistic infections might become more inspire to have well health and this might give energy to take the prescribed HAART appropriately. First-line HAART relatively has a less adverse effect than second-line HAART. Moreover, the level of adherence of children to first-line HAART could predict the probability of adherence to second-line HAART [68]. If children unfitted to first line HAART due to drug resistance and/or treatment failure, the probability they adhere with HAART could be less likely. The client might have enough information about the use of Cotrimoxazole prophylaxis so that they took the medication per the scheduled. Moreover, Cotrimoxazole prevent and control the occurrence and progression opportunistic infection that might help the child to be well adhered to HAART. On the other hand, protease inhibitors types of HAART [51] and caregivers not caring themselves [51] were significantly impair HAART adherence of children.

Our finding highlighted the importance of behavioral factors in HAART optimal adherence. Knowledge of caregiver [45, 46, 48, 49] was the most frequently reported factors positively associated with optimal adherence. Children whose caregiver was substance user [46, 55] and those didn’t use medication reminder [51] were negatively influencing HAART adherence. It is known that the use of alarming materials could remind the caregiver or children who forget the time of taking HAART so that they administer their medication as well. The association between optimal HAART adherence and disclosure of HIV status to children so far resulted differently [45–48]. This might be due to the finding relies on the caregiver report, which might not be accurately ascertained formal and unplanned disclosure status. In Ethiopia, by considering child’s psychological and social developmental status, sero-disclosure to children begins to be formal when they reach the age of 6 years and above. However, most of the studies included in this review wouldn’t consider these issues. Therefore, the finding from this review might provide an implication for further large scale study that concern about HIV disclosure status of children and HAART adherence in Ethiopia.

Our review found that proximity to the health care facility [46], and nutritional support [48] were promoting factors of optimal HAART adherence. As far as distance from health center increased, the probability of getting frequent information about the importance of HIV medication would decrease, which leads to a missed HAART doses. Furthermore, lack of vehicles and long distance might have a contribution to miss the appointment of HAART users. In our review, one study found that those children who received nutritional support [48] were more likely to have optimal HAART adherence. Nutritional support like plumy nut could help to maximize the health of children which aid to benefit the uses of HAART. Another studies out of Ethiopian settings also showed cost and access to transportation, lack of understanding of the benefit of HIV drugs, economic problems in the household, and lack of nutritional support have been associated with suboptimal HAART adherence [69–71].

Patient and HIV-care program monitoring, establish and strength linkages with other facility-based systems like TB monitoring, HIV care and HAART, HIV testing and counseling, perinatal care and electronic systems need to be implemented in every segments’ of Ethiopian settings. In addition, an improvement of healthcare settings, behavioral support, economic strengthening and home-based care indicated more emphasis.

## Strength and Limitation

This is the first systematic review and meta-analysis conducted in Ethiopia to show the national estimates of the prevalence of optimal adherence among HIV-infected children. There was no publication bias found in this meta-analysis as objectively explained by Egger’s regression test. It helps to increase the certainty of this evidence on decision making and resource utilization because the unbiased evidence is generated.

The reported past-three and 7 days adherence consider a relatively short window of period and may not represent adherence levels in the years. Since limited studies were conducted in some regions of the country, the current findings may not be nationally representative. High heterogeneity was found, as well as the scarcity of available factors to explain this variability only study setting was considered in the subgroup analysis. Another limitation is that all of the studies included in this systematic review and meta-analysis applied a cross-sectional design, making it difficult to determine the causal relationship between prevalence of optimal adherence and factors. Method of assessment of studies was care-giver report, which could overestimate adherence because of a desire to please the treatment provider and prevent criticism. Additionally, care-giver report could be vulnerable to recall-bias.

## Conclusion and Future Directions

Our study suggests that, within short window reported time, adherence to HAART in Ethiopian children may be in a good progress. This review revealed demographic characters, clinical and medication, behavioral and health care system related factors have been contributed on children HAART adherence status. The lowest prevalence of optimal adherence observed in Oromia region, Ethiopia. Emphasis on specific adherence interventions need further based on individual predictors to improve overall HAART adherence of children in Ethiopia.

## Electronic supplementary material

Below is the link to the electronic supplementary material.

Additional file Figure 1: Subgroup analysis 03 days prior to an interview. The midpoint and the length of each segment indicated prevalence and a 95% CI whereas the diamond shape showed the combined prevalence of all studies. Supplementary material 1 (PNG 22 kb)

Additional file Figure 2: Subgroup analysis 07 days prior to an interview time. The midpoint and the length of each segment indicated prevalence and a 95% CI whereas the diamond shape showed the combined prevalence of all studies. Supplementary material 2 (PNG 38 kb)
