# Supplementary figures and images for: Adherence to Highly Active Antiretroviral Therapy Among Children in Ethiopia: A Systematic Review and Meta-analysis
Source: AIDS Behav. 2018 May 14;22(8):2513–23. doi: 10.1007/s10461-018-2152-z (PMC6097744; doi:10.1007/s10461-018-2152-z)

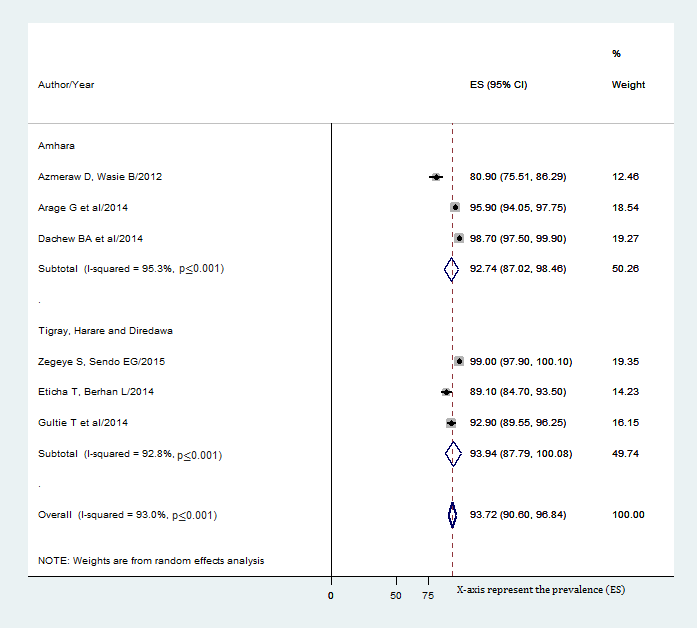

Supplement: Supplementary file 1 — Additional file Figure 1: Subgroup analysis 03 days prior to an interview. The midpoint and the length of each segment indicated prevalence and a 95% CI whereas the diamond shape showed the combined prevalence of all studies. Supplementary material 1 (PNG 22 kb) [file 10461_2018_2152_MOESM1_ESM.png]

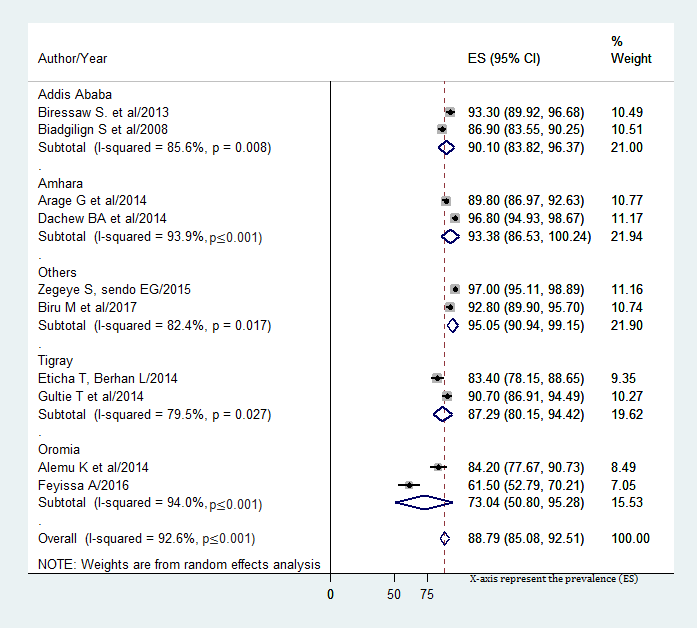

Supplement: Supplementary file 2 — Additional file Figure 2: Subgroup analysis 07 days prior to an interview time. The midpoint and the length of each segment indicated prevalence and a 95% CI whereas the diamond shape showed the combined prevalence of all studies. Supplementary material 2 (PNG 38 kb) [file 10461_2018_2152_MOESM2_ESM.png]
